# Meta-Analysis of the Association between Vitiligo and Human Leukocyte Antigen-A

**DOI:** 10.1155/2016/5412806

**Published:** 2016-09-05

**Authors:** Zhangjun Li, Jianwen Ren, Xinwu Niu, Qingqiang Xu, Xiaopeng Wang, Yale Liu, Shengxiang Xiao

**Affiliations:** ^1^Department of Dermatology, Second Affiliated Hospital of Xi'an Jiaotong University, Xi'an 710004, China; ^2^Department of Dermatology, Traditional Chinese Medicine Hospital of Shaanxi Province, Xi'an 710003, China

## Abstract

*Objective*. The objective of this study was to systematically evaluate the association between vitiligo and human leukocyte antigen- (HLA-) A.* Methods*. PubMed, Embase, Web of Science, Chinese National Knowledge Infrastructure, and reference lists were searched for relevant original articles.* Results*. Nineteen case-control studies comprising 3042 patients and 5614 controls were included, in which 33 HLA-A alleles were reported. Overall, three alleles (HLA-A^⁎^02, A^⁎^33, and Aw^⁎^31) were significantly associated with increased risk of vitiligo, two (HLA-A^⁎^09 and Aw^⁎^19) were associated with decreased risk, and the remaining 28 were unassociated. Twelve alleles, seven alleles, and 19 alleles were common to three ethnicities, both types of vitiligo, and both typing methods, respectively. In the subgroup analysis by ethnicity and typing methods, the association of six alleles and five alleles was inconsistent in three populations and both typing methods, respectively. In the subgroup analysis by clinical type, the association of all seven alleles was consistent in both types of vitiligo.* Conclusion*. The meta-analysis suggests that HLA-A^⁎^02, A^⁎^33, and Aw^⁎^31 are associated with increased risk of vitiligo, while HLA-A^⁎^09 and Aw^⁎^19 are associated with decreased risk of vitiligo. The association of some alleles varies in terms of ethnicity and typing methods.

## 1. Introduction

Vitiligo is an acquired depigmentation disorder of the skin characterized by absence of functional melanocytes. It affects approximately 0.5–2% of the world's population and impairs the patients' quality of life [1, 2]. The exact pathogenesis of vitiligo remains unknown; however, many potential theories have been proposed, including autoimmune, neural, genetic, melanocytorrhagy, and reactive oxygen species model hypotheses [3]. Among these, the autoimmune hypothesis is currently most widely accepted because of the frequent occurrence of other concomitant autoimmune diseases [4, 5] and the presence of circulating autoantibodies against pigment cells [6, 7]. Several genetic epidemiological studies have also demonstrated that genetic factors play an important role in the pathogenesis of vitiligo [8, 9].

The inherited nature of vitiligo and its frequent association with autoimmune diseases have prompted numerous studies on the association of vitiligo with human leukocyte antigens (HLAs), especially with HLA-A [10–26]. However, the results of these studies are controversial due to distinct ethnic populations, small sample sizes, and different research methods. With the development of molecular biology, genome-wide association studies have been successful in identifying susceptibility loci of vitiligo. Some authors have found that vitiligo is associated with HLA-A locus in Caucasians and the Japanese [27, 28]. A previous meta-analysis suggested that HLA-A2 was significantly associated with vitiligo [29], but the quality and strength of evidence were limited by the number of included studies. Moreover, newly published studies showed no association between vitiligo and HLA-A2 [10, 11, 13, 14].

Therefore, the objective of this meta-analysis was to systematically evaluate the association between vitiligo and HLA-A.

## 2. Methods

### 2.1. Search Strategy

Four electronic databases, PubMed, Embase, Web of Science, and Chinese National Knowledge Infrastructure (CNKI), were searched to screen all the case-control studies on the association between vitiligo and HLA, using free text and the Medical Subject Headings (MeSH) terms “vitiligo,” “human leukocyte antigen,” “HLA,” “major histocompatibility complex,” and “MHC.” The search period was from the start of each database up to February 2016, and articles were published in either English or Chinese. Moreover, reference lists from the retrieved articles were checked manually for additional studies.

### 2.2. Criteria for Inclusion and Exclusion

Studies were included if they met the following criteria: (1) primary studies exploring the association between vitiligo and HLA-A; (2) case-control design; (3) studies with full-text articles; (4) studies presenting sufficient data for calculating odds ratios (ORs); and (5) serological and molecular methods used for HLA-A typing. Exclusion criteria were as follows: (1) no original research (reviews, abstracts, editorials, case reports, and nonresearch letters); (2) studies without control subjects; (3) incomplete raw data; and (4) duplicate articles or reused data.

### 2.3. Data Extraction and Quality Assessment

Two investigators (Zhangjun Li and Jianwen Ren) independently extracted data from all eligible studies. Any disagreements were resolved by discussion and consensus with a third investigator (Shengxiang Xiao). The following data were recorded: first author, publication year, study design, country, ethnicity, characteristics of study population, numbers of cases and controls, typing methods, frequencies of HLA-A alleles, and study quality.

The methodological quality of included studies was assessed using the criteria proposed by Chalmers et al. [30], which consists of three major aspects: selection of subjects, comparability between groups, and outcome presented. The selected studies were rated on an ordinal star scoring scale from one to nine, with scores of five or more stars representing high quality [31].

### 2.4. Statistical Analysis

The chi-square and Fisher's exact tests were applied to compare the frequencies of HLA-A alleles in patients with vitiligo and controls to confirm the associated alleles, with significance set at *P* < 0.05. Meta-analysis of the association between HLA-A alleles and vitiligo was performed using two different approaches: a fixed effects model and a random effects model. Heterogeneity among studies was evaluated through the chi-square test and *I* ^2^ statistic, and *P* < 0.10 or *I* ^2^ > 50% was considered statistically significant. The pooled ORs and 95% confidence intervals (CIs) were calculated using either the random effects model when heterogeneity was confirmed or the fixed effects model when heterogeneity was absent. The test for overall effect was conducted using *Z*-scores, with significance set at *P* < 0.05. Subgroup analyses were conducted according to ethnicity, clinical type, and typing methods. Sensitivity was analyzed by omitting each study at each step to assess whether any single study had a significant influence on the pooled OR. Finally, publication bias was assessed by Begg's funnel plots and Egger's linear regression test, and the significance level was set at *P* < 0.05. All statistical analyses were performed using SPSS software (version 19.0; SPSS Institute, Chicago, USA) and STATA software (version 12.0; Stata Corporation, College Station, TX, USA). All tests were two-sided.

## 3. Results

### 3.1. Literature Search

**Figure 1:**
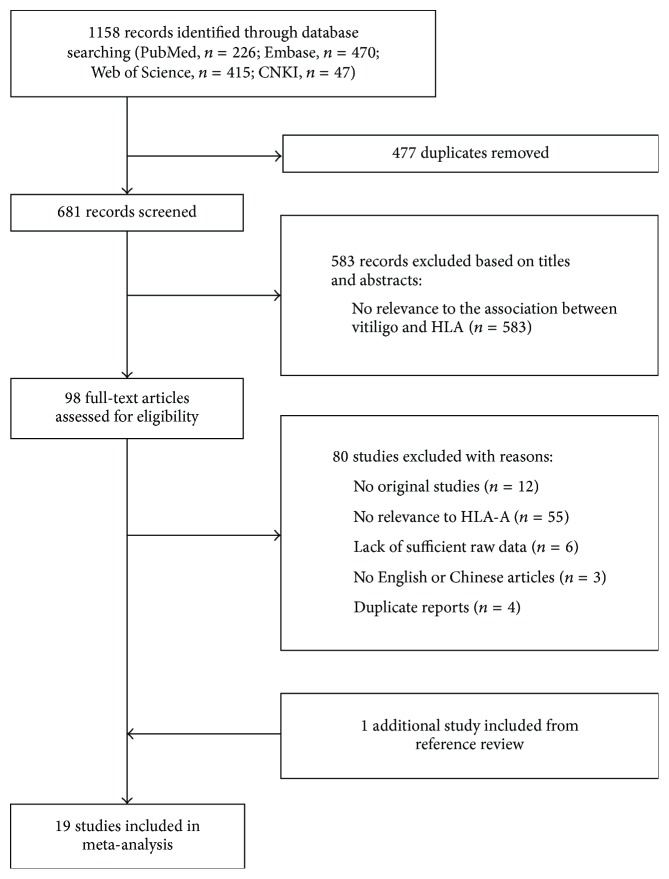
Flow diagram of study selection. HLA: human leukocyte antigen.

Initially, a total of 1158 records were identified through database searches. After removing duplicates and screening titles and abstracts, 98 full-text articles were reviewed and 18 studies [10–23, 25, 26, 32, 33] finally met the inclusion criteria. One additional study [24] was identified from a review of the reference lists. Altogether, 19 case-control studies were included in this meta-analysis. The procedure of literature search and study selection is shown in Figure 1.

### 3.2. Study Characteristics

The main characteristics of the included studies are summarized in Table 1. These 19 studies comprised 3042 patients with vitiligo and 5614 controls. Twelve studies [10–17, 19, 22, 24, 32] were conducted in Asians, four [18, 20, 21, 23] were performed in Europeans, two [26, 33] were investigated in Americans, and the remaining one [25] was carried out in mixed populations. HLA-A typing methods such as lymphocytotoxicity test (LCT) [11, 13, 15–26], polymerase chain reaction sequence-specific oligonucleotides (PCR-SSO) [33], PCR sequence-specific oligonucleotide probes (PCR-SSOP) [32], and PCR sequence-specific primers (PCR-SSP) [10, 12, 14] were reported in the studies. In total, 33 HLA-A alleles were involved. The results of the chi-square and Fisher's exact tests indicated that 18 alleles were associated with vitiligo and 31 were unassociated. Sixteen alleles were disputed. According to the quality assessment criteria [30, 31], all the 19 studies [10–26, 32, 33] were of high quality with scores between five and nine stars.

### 3.3. Association between Vitiligo and Human Leukocyte Antigen-A

**Figure 2:**
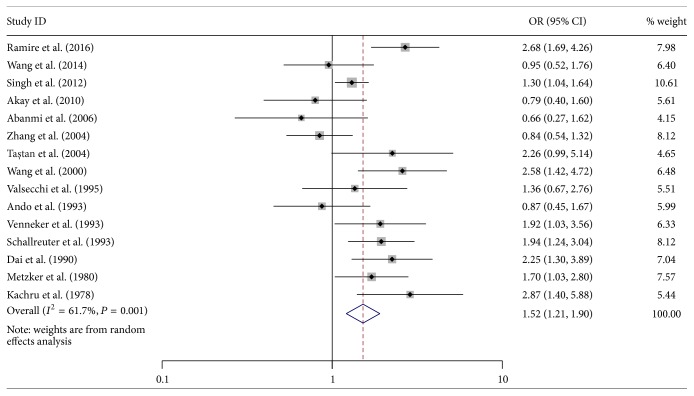
Forest plot of 15 included studies on the association between vitiligo and human leukocyte antigen- (HLA-) A^*∗*^02. OR: odds ratio; CI: confidence interval.

The general information on the association of vitiligo with HLA-A is given in Table 2. Among the 33 HLA-A alleles included in the pooled analysis, three (HLA-A^*∗*^02, A^*∗*^33, and Aw^*∗*^31) were significantly associated with increased risk of vitiligo, while two (HLA-A^*∗*^09 and Aw^*∗*^19) were associated with decreased risk. HLA-A^*∗*^02 was reported in 15 studies. The pooled OR calculated with the random effects model was 1.52 (95% CI: 1.21–1.90, *P* < 0.001) (Figure 2), and the heterogeneity was significant (*I* ^2^ = 61.7%, *P* = 0.001).

**Figure 3:**
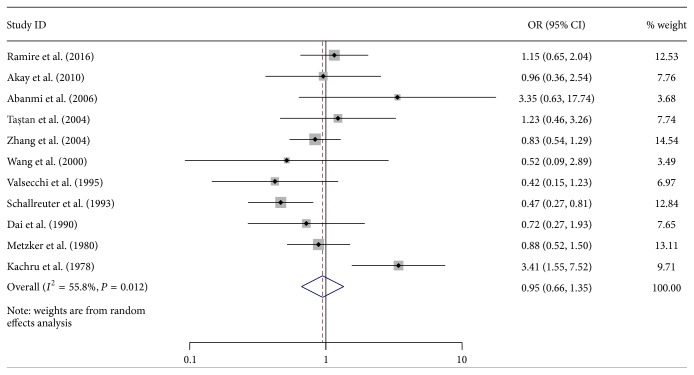
Forest plot of 11 included studies on the association between vitiligo and human leukocyte antigen- (HLA-) A^*∗*^01. OR: odds ratio; CI: confidence interval.

**Figure 4:**
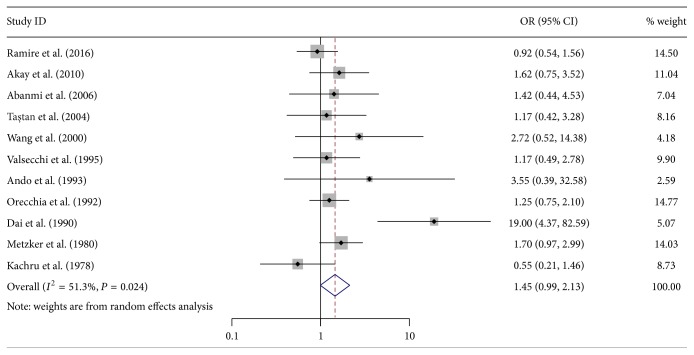
Forest plot of 11 included studies on the association between vitiligo and human leukocyte antigen- (HLA-)  A^*∗*^03. OR: odds ratio; CI: confidence interval.

The rest 28 alleles were not associated with vitiligo, of which HLA-A^*∗*^01 and A^*∗*^03 were each involved in more than 10 studies. The pooled ORs calculated with the random effects model were 0.95 (95% CI: 0.66–1.35, *P* = 0.761) and 1.45 (95% CI: 0.99–2.13, *P* = 0.056), respectively (Figures 3 and 4). Significant heterogeneity among the studies was found (*I* ^2^ = 55.8%, *P* = 0.012, and *I* ^2^ = 51.3%, *P* = 0.024, resp.).

### 3.4. Subgroup Analysis according to Ethnicity

Table 3 presents the results of subgroup analysis based on ethnicity. Of the 26 HLA-A alleles studied in Asian patients with vitiligo, four (HLA-A^*∗*^03, A^*∗*^10, A^*∗*^25, and A^*∗*^33) were significantly associated with increased risk of vitiligo and three (HLA-A^*∗*^09, A^*∗*^66, and Aw^*∗*^19) were associated with decreased risk. The remaining 19 alleles were not associated with vitiligo. Among the 12 HLA-A alleles reported in European cases, one (HLA-A^*∗*^02) was significantly associated with increased risk of vitiligo, and one (HLA-A^*∗*^01) was associated with decreased risk. The remaining 10 alleles were not associated. For American cases, 29 HLA-A alleles were studied. Two alleles (HLA-A^*∗*^02 and Aw^*∗*^31) were significantly associated with increased risk of vitiligo, and one (HLA-A^*∗*^10) was associated with decreased risk. The other 26 alleles were not associated.

Twelve alleles (HLA-A^*∗*^01, A^*∗*^02, A^*∗*^03, A^*∗*^09, A^*∗*^10, A^*∗*^11, A^*∗*^28, A^*∗*^29, A^*∗*^30, A^*∗*^31, A^*∗*^32, and A^*∗*^33) were common to Asians, Europeans, and Americans, but six (HLA-A^*∗*^01, A^*∗*^02, A^*∗*^03, A^*∗*^09, A^*∗*^10, and A^*∗*^33) of them were inconsistent in their association with vitiligo.

### 3.5. Subgroup Analysis according to Clinical Type

Table 4 demonstrates the results of subgroup analysis based on clinical type. Among the 26 HLA-A alleles studied in patients with nonsegmental vitiligo, three (HLA-A^*∗*^02, A^*∗*^03, and A^*∗*^33) were significantly associated with increased risk of nonsegmental vitiligo and one (HLA-A^*∗*^19) was associated with decreased risk. The remaining 22 alleles were not associated with nonsegmental vitiligo. Of the seven HLA-A alleles reported in cases of segmental vitiligo, two (HLA-A^*∗*^02 and A^*∗*^03) were significantly associated with increased risk of segmental vitiligo and the remaining five were not associated.

Seven alleles (HLA-A^*∗*^01, A^*∗*^02, A^*∗*^03, A^*∗*^09, A^*∗*^10, A^*∗*^11, and A^*∗*^28) were common to both types of vitiligo. Moreover, all of them were consistent in their association with vitiligo.

### 3.6. Subgroup Analysis according to Typing Methods

Table 5 indicates the results of subgroup analysis based on typing methods. Two kinds of HLA-A typing methods were involved: serological methods (LCT) and molecular methods (PCR-SSO, PCR-SSOP, and PCR-SSP). Of the 31 HLA-A alleles detected by serological methods, four (HLA-A^*∗*^02, A^*∗*^03, A^*∗*^31, and Aw^*∗*^31) were significantly associated with increased risk of vitiligo and one (HLA-A^*∗*^09) was associated with decreased risk. The remaining 26 alleles were not associated with vitiligo. Among the 21 HLA-A alleles detected by molecular methods, two (HLA-A^*∗*^36 and Aw^*∗*^19) were significantly associated with increased risk of vitiligo and the remaining 19 were not associated.

Nineteen alleles (HLA-A^*∗*^01, A^*∗*^02, A^*∗*^03, A^*∗*^11, A^*∗*^23, A^*∗*^24, A^*∗*^25, A^*∗*^26, A^*∗*^29, A^*∗*^30, A^*∗*^31, A^*∗*^32, A^*∗*^33, A^*∗*^36, A^*∗*^43, A^*∗*^66, A^*∗*^68, A^*∗*^80, and Aw^*∗*^19) were common to both typing methods. However, five (HLA-A^*∗*^02, A^*∗*^03, A^*∗*^31, A^*∗*^36, and Aw^*∗*^19) of them were inconsistent in their association with vitiligo.

### 3.7. Sensitivity Analysis and Publication Bias

**Figure 5:**
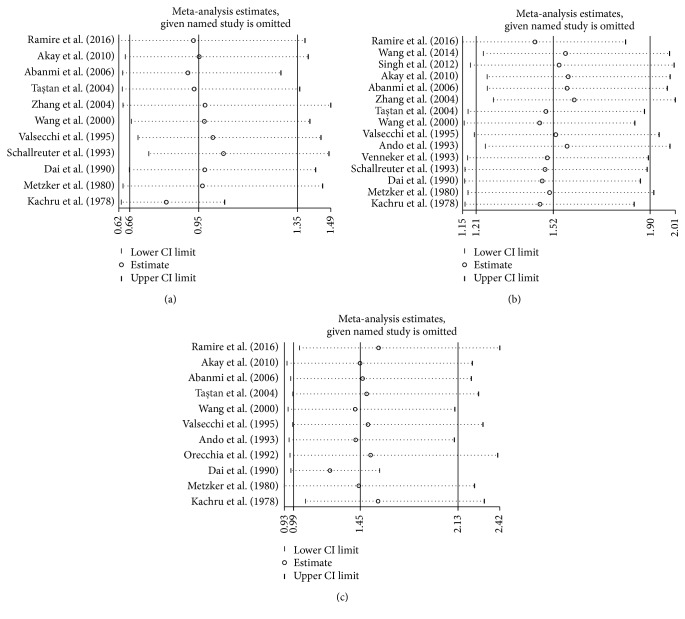
Influence of removing individual studies on adjusted effect estimates of (a) human leukocyte antigen- (HLA-) A^*∗*^01, (b) A^*∗*^02, and (c) A^*∗*^03. Circles are effect estimates and horizontal dotted lines are 95% confidence intervals (CIs) for meta-analysis of the remaining studies. Vertical lines in the centers are the pooled effect estimates for all studies, respectively.

**Figure 6:**
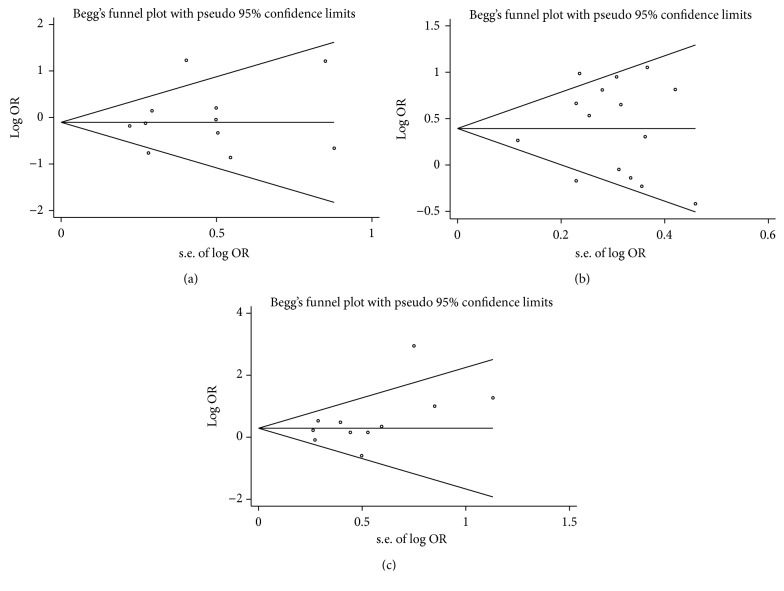
Funnel plots for meta-analysis of the association of vitiligo with (a) human leukocyte antigen- (HLA-) A^*∗*^01, (b) A^*∗*^02, and (c) A^*∗*^03. log OR: natural logarithm of odds ratio.

Among the 33 alleles investigated in 19 included studies, HLA-A^*∗*^01, A^*∗*^02, and A^*∗*^03 were reported in more than 10 studies and were therefore chosen for sensitivity analysis and assessment of publication bias. As shown in Figure 5, sensitivity analyses indicated that no single study substantially influenced the pooled ORs qualitatively (data not shown). Begg's funnel plots and Egger's test were performed to assess publication bias. No obvious publication bias was found in the results (HLA-A^*∗*^01, A^*∗*^02, and A^*∗*^03: *P* = 0.566, 0.749, and 0.160, resp.) (Figure 6).

## 4. Discussion

In the present study, we performed a meta-analysis to comprehensively evaluate the association between vitiligo and 33 HLA-A alleles. Nineteen case-control studies [10–26, 32, 33] with a total of 3042 vitiligo cases and 5614 controls were finally identified from four databases and reference review. Overall, three alleles (HLA-A^*∗*^02, A^*∗*^33, and Aw^*∗*^31) were significantly associated with increased risk of vitiligo, while two (HLA-A^*∗*^09 and Aw^*∗*^19) were associated with decreased risk. The association between HLA-A^*∗*^02 and vitiligo was consistent with the results of Liu et al. [29]. In addition, the remaining 28 alleles were not associated with vitiligo.

There were 12 alleles common to three ethnicities (Asians, Europeans, and Americans), seven alleles common to both types of vitiligo (nonsegmental and segmental), and 19 alleles common to both typing methods (serological and molecular). In the subgroup analysis by ethnicity, the association of six alleles was consistent in three populations, while that of the remaining six alleles (HLA-A^*∗*^01, A^*∗*^02, A^*∗*^03, A^*∗*^09, A^*∗*^10, and A^*∗*^33) was inconsistent. The possible reasons for these inconsistencies might be the difference in ethnicity or the comparatively small number of included studies for some alleles. Subgroup analysis by clinical type showed that the association of all seven alleles (HLA-A^*∗*^01, A^*∗*^02, A^*∗*^03, A^*∗*^09, A^*∗*^10, A^*∗*^11, and A^*∗*^28) was consistent in both types of vitiligo. It suggests that the association between vitiligo and these alleles may be independent of clinical type. However, we should interpret this association with great caution because only two studies [16, 24] presented relevant data on segmental vitiligo and were included in this meta-analysis. HLA-A typing is critical for the accuracy of test results. In the 19 studies of this meta-analysis, serological and molecular methods were involved, and the latter had higher resolution than the former. In the subgroup analysis by typing methods, the association of five alleles (HLA-A^*∗*^02, A^*∗*^03, A^*∗*^31, A^*∗*^36, and Aw^*∗*^19) was inconsistent in both typing methods. It suggests that the association of vitiligo with these five alleles may vary in terms of typing methods.

Among the 33 HLA-A alleles in the current meta-analysis, only three alleles (HLA-A^*∗*^01, A^*∗*^02, and A^*∗*^03) were reported in more than 10 studies. There was obvious heterogeneity among the studies for each allele, which might be caused by the differences in ethnicity, clinical type, and typing methods. However, sensitivity analyses indicated that the results for these three alleles were statistically reliable, and no publication bias was found based on the funnel plot analyses and Egger's tests. The association between the remaining 30 alleles and vitiligo needs to be further studied.

This study has some limitations. First, the meta-analysis only included published studies. Second, vitiligo may be influenced by not only genetic factors but also environmental factors. The results of the meta-analysis should be interpreted cautiously owing to the lack of available data regarding vitiligo development and its relationship with genetic and environmental factors. Further studies may assess the possible gene-environment interactions in the association. Third, the relatively small samples of some HLA-A alleles limited the statistical power. Finally, we were not able to perform subgroup for each HLA-A allele due to the limited number of eligible studies, which might have affected the results. Therefore, more studies with larger sample sizes focusing on each HLA-A allele are needed to confirm these findings. Despite the limitations listed above, this study still has some strength. To the best of our knowledge, this is the first meta-analysis evaluating the association between vitiligo and a number of HLA-A alleles.

## 5. Conclusion

In summary, this meta-analysis suggests that HLA-A^*∗*^02, A^*∗*^33, and Aw^*∗*^31 are associated with increased risk of vitiligo, while HLA-A^*∗*^09 and Aw^*∗*^19 are associated with decreased risk of vitiligo. Moreover, the association of some alleles varies in terms of ethnicity and typing methods. However, further well-designed studies with larger sample sizes are still needed to confirm our findings.

## Figures and Tables

**Table 1:** Characteristics of studies included in the meta-analysis.

Study	Country	Ethnicity	Patients		Controls		Typing methods	Significant association with HLA-A (*P* < 0.05)		Quality score
			Clinical types	Number	Types	Number		Positive	Negative	
Ramire et al., 2016 [33]	Brazil	American	27 localized, 78 generalized, 11 nonclassified	116	NCs	243	PCR-SSO	A02, 25, 34	A01, 03, 11, 23, 24, 26, 29, 30, 31, 32, 33, 36, 66, 68, 69, 80	8
Wang et al., 2014 [10]	China	Asian	Unknown	97	NCs	72	PCR-SSP		A02	7
Singh et al., 2012 [32]	India	Asian	411 localized, 1347 generalized	1758	NCs	1310	PCR-SSOP	A02, 26, 31, 33	A68	7
Akay et al., 2010 [11]	Turkey	Asian	Unknown	52	NCs	100	LCT	A24, 30	A01, 02, 03, 04, 09, 10, 11, 23, 25, 26, 29, 32, 66, 68	9
Wang et al., 2007 [12]	China	Asian	34 generalized	34	NCs	102	PCR-SSP	A30		9
Abanmi et al., 2006 [13]	Saudi Arabia	Asian	34 generalized, 6 universal	40	NCs	40	LCT	A09	A01, 02, 03, 10, 11, 19, 28, 36, 38, 43, 80	9
Zhang et al., 2004 [14]	China	Asian	93 localized, 94 generalized	187	NCs	252	PCR-SSP	A25, 30, 31, 36, 66	A01, 02, 26, 43	9
Taştan et al., 2004 [15]	Turkey	Asian	Unknown	33	NCs	100	LCT	A24	A01, 02, 03, 11, 23, 26, 29, 30, 31, 32, 33, 68	5
Wang et al., 2000 [16]	China	Asian	40 vulgaris, 22 focal, 8 acrofacial, 25 segmental	95	NCs	100	LCT	A02, 10, 28	A01, 03, 09, 11	9
Valsecchi et al., 1995 [18]	Italy	European	20 vulgaris, 3 focal, 7 acrofacial, 3 acral	33	NCs	443	LCT		A01, 02, 03, 09, 10, 11, 28, 29, 30, 31, 32, 33	6
Venkataram et al., 1995 [17]	Oman	Asian	29 focal, 21 acrofacial	50	NCs	92	LCT		A30	7
Al-Fouzan et al., 1995 [19]	Kuwait	Asian	40 nonsegmental	40	NCs	40	LCT	A19		9
Venneker et al., 1993 [20]	Dutch	European	48 generalized	48	NCs	703	LCT		A02	6
Schallreuter et al., 1993 [21]	German	European	57 vulgaris, 13 focal, 22 acrofacial, 7 universal, 3 segmental	102	NCs	400	LCT	A01, 02		9
Ando et al., 1993 [22]	Japan	Asian	39 nonsegmental	39	NCs	544	LCT		A02, 03, 11, 24, 26, 31, Aw33	5
Orecchia et al., 1992 [23]	Italy	European	65 vulgaris, 13 focal, 7 acrofacial, 8 acral	93	NCs	388	LCT	A30	A03, 09	6
Dai et al., 1990 [24]	China	Asian	30 focal, 40 generalized, 30 segmental	100	NCs	116	LCT	A02, 03	A01, 09, 10, 11, 28	6
Metzker et al., 1980 [25]	Israel	Mixed	Unknown	77	NCs	462	LCT	A02, 11	A01, 03, 09, 10, 28, 29, Aw19	5
Kachru et al., 1978 [26]	America	American	Unknown	48	NCs	107	LCT	A01, 02, 10, Aw31	A03, 09, 11, 28, 29, Aw19, 23, 24, 30, 32, 33	7

HLA: human leukocyte antigen, Number: number of subjects, NCs: normal controls, LCT: lymphocytotoxicity test, PCR-SSP: polymerase chain reaction sequence-specific primers, PCR-SSO: polymerase chain reaction sequence-specific oligonucleotides, and PCR-SSOP: polymerase chain reaction sequence-specific oligonucleotide probes.

**Table 2:** Association between vitiligo and 33 human leukocyte antigen- (HLA-) A alleles.

Allele	Patients% (*n*/*N*)	Controls% (*n*/*N*)	OR (95% CI)/article number	*P*
A^*∗*^01	28.20 (249/883)	28.65 (677/2363)	0.95r (0.66, 1.35)/11	0.761
A^*∗*^02	26.94 (761/2825)	34.25 (1710/4992)	1.52r (1.21, 1.90)/15	<0.001
A^*∗*^03	19.70 (143/726)	14.30 (378/2643)	1.45r (0.99, 2.13)/11	0.056
A^*∗*^04	3.85 (2/52)	3.00 (3/100)	1.29 (0.21, 7.99)/1	0.782
A^*∗*^09	20.07 (108/538)	26.25 (461/1756)	0.70 (0.54, 0.90)/8	0.005
A^*∗*^10	10.56 (47/445)	12.79 (175/1368)	1.11r (0.51, 2.41)/7	0.786
A^*∗*^11	15.80 (100/633)	14.41 (325/2255)	0.87 (0.66, 1.14)/10	0.306
A^*∗*^19	22.50 (18/80)	43.75 (35/80)	0.19r (0.02, 2.02)/2	0.170
A^*∗*^23	5.47 (11/201)	7.22 (32/443)	0.76 (0.38, 1.53)/3	0.436
A^*∗*^24	21.67 (52/240)	40.53 (400/987)	0.89r (0.36, 2.21)/4	0.800
A^*∗*^25	11.83 (42/355)	5.71 (34/595)	1.29r (0.26, 6.43)/3	0.756
A^*∗*^26	8.05 (176/2185)	13.42 (342/2549)	0.79r (0.53, 1.19)/6	0.265
A^*∗*^28	7.89 (31/393)	8.60 (109/1268)	0.74 (0.46, 1.17)/6	0.192
A^*∗*^29	6.13 (22/359)	7.90 (115/1455)	0.78 (0.49, 1.27)/6	0.320
A^*∗*^30	12.37 (74/598)	6.92 (119/1720)	1.83r (0.88, 3.81)/8	0.107
A^*∗*^31	3.88 (84/2166)	6.54 (189/2892)	1.33r (0.61, 2.90)/6	0.479
A^*∗*^32	6.41 (15/234)	8.24 (73/886)	0.78 (0.43, 1.40)/4	0.400
A^*∗*^33	22.16 (430/1940)	8.78 (184/2096)	2.23 (1.84, 2.70)/4	<0.001
A^*∗*^34	0.00 (0/116)	3.29 (8/243)	0.12 (0.01, 2.08)/1	0.145
A^*∗*^36	0.58 (2/343)	2.24 (12/535)	0.43r (0.04, 5.02)/3	0.501
A^*∗*^38	0.00 (0/40)	2.50 (1/40)	0.33 (0.01, 8.22)/1	0.495
A^*∗*^43	31.72 (72/227)	36.64 (107/292)	0.85 (0.58, 1.24)/2	0.394
A^*∗*^66	2.82 (10/355)	7.73 (46/595)	0.58r (0.08, 4.25)/3	0.595
A^*∗*^68	14.45 (283/1959)	12.09 (212/1753)	1.12 (0.93, 1.36)/4	0.238
A^*∗*^69	0.00 (0/116)	0.82 (2/243)	0.41 (0.02, 8.71)/1	0.571
A^*∗*^80	1.28 (2/156)	1.77 (5/283)	0.52 (0.11, 2.43)/2	0.405
Aw^*∗*^19	0.72 (13/1806)	6.35 (90/1417)	0.11 (0.06, 0.19)/2	<0.001
Aw^*∗*^23	2.08 (1/48)	4.67 (5/107)	0.43 (0.05, 3.82)/1	0.452
Aw^*∗*^24	0.00 (0/48)	2.80 (3/107)	0.31 (0.02, 6.08)/1	0.439
Aw^*∗*^30	20.83 (10/48)	28.04 (30/107)	0.68 (0.30, 1.52)/1	0.345
Aw^*∗*^31	16.67 (8/48)	4.67 (5/107)	4.08 (1.26, 13.22)/1	0.019
Aw^*∗*^32	4.17 (2/48)	8.41 (9/107)	0.47 (0.10, 2.28)/1	0.351
Aw^*∗*^33	9.20 (8/87)	12.29 (80/651)	1.03 (0.46, 2.30)/1	0.944

*N*: total number of subjects, *n*: positive number of subjects, OR: odds ratio, CI: confidence interval, article number: total number of the articles relevant to the association between vitiligo and HLA-A alleles, r: random effects model, and the others: fixed effects model.

**Table 3:** Association between vitiligo and human leukocyte antigen- (HLA-) A alleles in terms of ethnicity.

Ethnicity	Allele	Patients% (*n*/*N*)	Controls% (*n*/*N*)	OR (95% CI)/article number	*P*
Asian	A^*∗*^01	32.54 (165/507)	34.04 (241/708)	0.91 (0.66, 1.26)/6	0.574
	A^*∗*^02	21.45 (515/2401)	26.31 (693/2634)	1.24r (0.93, 1.67)/9	0.147
	A^*∗*^03	16.71 (60/359)	5.00 (50/1000)	2.46r (1.11, 5.45)/6	0.026
	A^*∗*^04	3.85 (2/52)	3.00 (3/100)	1.29 (0.21, 7.99)/1	0.782
	A^*∗*^09	18.82 (54/287)	24.72 (88/356)	0.60 (0.40, 0.89)/4	0.010
	A^*∗*^10	10.45 (30/287)	3.65 (13/356)	2.73 (1.40, 5.35)/4	0.003
	A^*∗*^11	22.84 (82/359)	20.10 (201/1000)	0.99 (0.71, 1.38)/6	0.960
	A^*∗*^19	22.50 (18/80)	43.75 (35/80)	0.19r (0.02, 2.02)/2	0.170
	A^*∗*^23	2.35 (2/85)	4.00 (8/200)	0.72 (0.17, 3.11)/2	0.662
	A^*∗*^24	25.00 (31/124)	48.12 (358/744)	0.79r (0.17, 3.76)/3	0.767
	A^*∗*^25	17.57 (42/239)	7.10 (25/352)	2.65 (1.56, 4.51)/2	<0.001
	A^*∗*^26	8.07 (167/2069)	13.96 (322/2306)	0.76r (0.47, 1.24)/5	0.274
	A^*∗*^28	7.66 (18/235)	11.33 (29/256)	0.43r (0.08, 2.20)/3	0.311
	A^*∗*^29	2.35 (2/85)	5.00 (10/200)	0.45 (0.10, 2.08)/2	0.307
	A^*∗*^30	13.20 (47/356)	6.50 (42/646)	2.18r (0.85, 5.61)/5	0.107
	A^*∗*^31	3.72 (75/2017)	6.53 (144/2206)	1.79r (0.55, 5.85)/4	0.335
	A^*∗*^32	8.24 (7/85)	9.00 (18/200)	0.90 (0.36, 2.23)/2	0.813
	A^*∗*^33	23.56 (422/1791)	11.28 (159/1410)	2.32 (1.90, 2.83)/2	<0.001
	A^*∗*^36	0.89 (2/227)	2.74 (8/292)	0.61r (0.01, 40.53)/2	0.818
	A^*∗*^38	0.00 (0/40)	2.50 (1/40)	0.33 (0.01, 8.22)/1	0.495
	A^*∗*^43	31.72 (72/227)	36.64 (107/292)	0.85 (0.58, 1.24)/2	0.394
	A^*∗*^66	2.93 (7/239)	12.50 (44/352)	0.20 (0.09, 0.45)/2	<0.001
	A^*∗*^68	14.38 (265/1843)	12.58 (190/1510)	1.08 (0.88, 1.32)/3	0.476
	A^*∗*^80	5.00 (2/40)	10.00 (4/40)	0.47 (0.08, 2.75)/1	0.405
	Aw^*∗*^19	0.74 (13/1758)	6.79 (89/1310)	0.10 (0.06, 0.18)/1	<0.001
	Aw^*∗*^33	12.82 (5/39)	13.79 (75/544)	0.92 (0.35, 2.43)/1	0.866

European	A^*∗*^01	16.30 (22/135)	27.88 (235/843)	0.46 (0.28, 0.74)/2	0.002
	A^*∗*^02	62.84 (115/183)	41.01 (757/1546)	1.80 (1.30, 2.48)/3	<0.001
	A^*∗*^03	25.40 (32/126)	20.58 (171/831)	1.23 (0.79, 1.92)/2	0.359
	A^*∗*^09	20.63 (26/126)	24.43 (203/831)	0.79 (0.50, 1.26)/2	0.321
	A^*∗*^10	9.09 (3/33)	13.09 (58/443)	0.66 (0.20, 2.25)/1	0.510
	A^*∗*^11	15.15 (5/33)	9.71 (43/443)	1.66 (0.61, 4.53)/1	0.321
	A^*∗*^28	9.09 (3/33)	5.42 (24/443)	1.75 (0.50, 6.13)/1	0.385
	A^*∗*^29	9.09 (3/33)	7.45 (33/443)	1.24 (0.36, 4.29)/1	0.731
	A^*∗*^30	9.52 (12/126)	4.81 (40/831)	1.70r (0.16, 18.02)/2	0.660
	A^*∗*^31	9.09 (3/33)	4.97 (22/443)	1.91 (0.54, 6.76)/1	0.313
	A^*∗*^32	12.12 (4/33)	7.90 (35/443)	1.61 (0.53, 4.83)/1	0.398
	A^*∗*^33	6.06 (2/33)	2.26 (10/443)	2.79 (0.59, 13.31)/1	0.197

American	A^*∗*^01	24.39 (40/164)	16.29 (57/350)	1.91r (0.66, 5.53)/2	0.230
	A^*∗*^02	60.98 (100/164)	36.86 (129/350)	2.73 (1.85, 4.03)/2	<0.001
	A^*∗*^03	18.90 (31/164)	22.29 (78/350)	0.81 (0.51, 1.29)/2	0.373
	A^*∗*^09	20.83 (10/48)	18.69 (20/107)	1.14 (0.49, 2.68)/1	0.775
	A^*∗*^10	8.33 (4/48)	27.10 (29/107)	0.24 (0.08, 0.74)/1	0.013
	A^*∗*^11	6.71 (11/164)	10.29 (36/350)	0.82r (0.20, 3.32)/2	0.776
	A^*∗*^23	7.76 (9/116)	9.88 (24/243)	0.77 (0.34, 1.71)/1	0.517
	A^*∗*^24	18.10 (21/116)	17.28 (42/243)	1.06 (0.59, 1.89)/1	0.849
	A^*∗*^25	0.00 (0/116)	3.70 (9/243)	0.11 (0.01, 1.84)/1	0.123
	A^*∗*^26	7.76 (9/116)	8.23 (20/243)	0.94 (0.41, 2.13)/1	0.878
	A^*∗*^28	6.25 (3/48)	11.21 (12/107)	0.53 (0.14, 1.96)/1	0.340
	A^*∗*^29	6.10 (10/164)	9.43 (33/350)	0.64 (0.31, 1.31)/2	0.222
	A^*∗*^30	12.93 (15/116)	15.23 (37/243)	0.83 (0.43, 1.58)/1	0.564
	A^*∗*^31	5.17 (6/116)	9.47 (23/243)	0.52 (0.21, 1.32)/1	0.169
	A^*∗*^32	3.45 (4/116)	8.23 (20/243)	0.40 (0.13, 1.19)/1	0.100
	A^*∗*^33	5.17 (6/116)	6.17 (15/243)	0.83 (0.31, 2.20)/1	0.706
	A^*∗*^34	0.00 (0/116)	3.29 (8/243)	0.12 (0.01, 2.08)/1	0.145
	A^*∗*^36	0.00 (0/116)	1.65 (4/243)	0.23 (0.01, 4.28)/1	0.323
	A^*∗*^66	2.59 (3/116)	0.82 (2/243)	3.20 (0.53, 19.41)/1	0.206
	A^*∗*^68	15.52 (18/116)	9.05 (22/243)	1.85 (0.95, 3.59)/1	0.072
	A^*∗*^69	0.00 (0/116)	0.82 (2/243)	0.41 (0.02, 8.71)/1	0.571
	A^*∗*^80	0.00 (0/116)	0.41 (1/243)	0.69 (0.03, 17.16)/1	0.823
	Aw^*∗*^19	0.00 (0/48)	0.93 (1/107)	0.73 (0.03, 18.29)/1	0.849
	Aw^*∗*^23	2.08 (1/48)	4.67 (5/107)	0.43 (0.05, 3.82)/1	0.452
	Aw^*∗*^24	0.00 (0/48)	2.80 (3/107)	0.31 (0.02, 6.08)/1	0.439
	Aw^*∗*^30	20.83 (10/48)	28.04 (30/107)	0.68 (0.30, 1.52)/1	0.345
	Aw^*∗*^31	16.67 (8/48)	4.67 (5/107)	4.08 (1.26, 13.22)/1	0.019
	Aw^*∗*^32	4.17 (2/48)	8.41 (9/107)	0.47 (0.10, 2.28)/1	0.351
	Aw^*∗*^33	6.25 (3/48)	4.67 (5/107)	1.36 (0.31, 5.94)/1	0.683

*N*: total number of subjects, *n*: positive number of subjects, OR: odds ratio, CI: confidence interval, article number: total number of the articles relevant to the association between vitiligo and HLA-A alleles, r: random effects model, and the others: fixed effects model.

**Table 4:** Association between vitiligo and human leukocyte antigen- (HLA-) A alleles in terms of clinical type.

Clinical type	Allele	Patients% (*n*/*N*)	Controls% (*n*/*N*)	OR (95% CI)/article number	*P*
Nonsegmental	A^*∗*^01	29.84 (94/315)	32.45 (355/1094)	0.81 (0.57, 1.14)/5	0.226
	A^*∗*^02	23.36 (425/1819)	33.08 (1241/3751)	1.46r (1.01, 2.10)/9	0.042
	A^*∗*^03	20.09 (85/423)	12.86 (241/1874)	1.98r (1.02, 3.81)/7	0.042
	A^*∗*^09	21.19 (50/236)	25.84 (255/987)	0.70 (0.49, 1.00/4	0.052
	A^*∗*^10	13.62 (29/213)	10.01 (70/699)	2.09r (0.83, 5.28)/4	0.117
	A^*∗*^11	16.15 (42/260)	15.73 (218/1386)	0.90 (0.61, 1.34)/5	0.607
	A^*∗*^19	22.50 (18/80)	43.75 (35/80)	0.28 (0.13, 0.63)/2	0.002
	A^*∗*^23	5.13 (4/78)	9.88 (24/243)	0.49 (0.17, 1.47)/1	0.204
	A^*∗*^24	25.64 (30/117)	46.25 (364/787)	0.72 (0.44, 1.17)/2	0.180
	A^*∗*^25	19.19 (33/172)	6.46 (32/495)	1.18r (0.03, 47.18)/2	0.930
	A^*∗*^26	7.64 (119/1558)	13.32 (313/2349)	0.91r (0.58, 1.43)/4	0.676
	A^*∗*^28	13.29 (19/143)	7.35 (44/599)	1.00 (0.50, 1.98)/3	0.996
	A^*∗*^29	7.21 (8/111)	8.60 (59/686)	0.75 (0.34, 1.62)/2	0.460
	A^*∗*^30	13.87 (53/382)	7.24 (110/1520)	1.84r (0.73, 4.62)/6	0.196
	A^*∗*^31	4.02 (64/1591)	6.66 (186/2792)	1.23r (0.54, 2.79)/5	0.626
	A^*∗*^32	3.60 (4/111)	8.02 (55/686)	0.41r (0.01, 15.55)/2	0.628
	A^*∗*^33	23.05 (336/1458)	9.02 (180/1996)	2.34 (1.91, 2.86)/3	<0.001
	A^*∗*^34	0.00 (0/78)	3.29 (8/243)	0.18 (0.01, 3.09)/1	0.235
	A^*∗*^36	0.94 (2/212)	2.24 (12/535)	0.54 (0.15, 1.94)/3	0.344
	A^*∗*^38	0.00 (0/40)	2.50 (1/40)	0.33 (0.01, 8.22)/1	0.495
	A^*∗*^43	25.37 (34/134)	36.64 (107/292)	0.75 (0.47, 1.22)/2	0.251
	A^*∗*^66	3.49 (6/172)	8.89 (44/495)	0.83r (0.03, 23.79)/2	0.914
	A^*∗*^68	16.07 (229/1425)	13.33 (207/1553)	1.21 (0.98, 1.48)/2	0.072
	A^*∗*^69	0.00 (0/78)	0.82 (2/243)	0.62 (0.03, 12.95)/1	0.755
	A^*∗*^80	1.69 (2/118)	1.77 (5/283)	0.56 (0.12, 2.66)/2	0.468
	Aw^*∗*^33	12.82 (5/39)	13.79 (75/544)	0.92 (0.35, 2.43)/1	0.866

Segmental	A^*∗*^01	6.67 (2/30)	9.48 (11/116)	0.68 (0.14, 3.26)/1	0.631
	A^*∗*^02	67.27 (37/55)	47.22 (102/216)	2.34 (1.25, 4.38)/2	0.008
	A^*∗*^03	7.27 (4/55)	1.85 (4/216)	4.09 (1.08, 15.51)/2	0.038
	A^*∗*^09	26.67 (8/30)	32.76 (38/116)	0.75 (0.30, 1.83)/1	0.523
	A^*∗*^10	7.27 (4/55)	3.70 (8/216)	2.03 (0.59, 7.01)/2	0.261
	A^*∗*^11	33.33 (10/30)	30.17 (35/116)	1.16 (0.49, 2.72)/1	0.738
	A^*∗*^28	6.67 (2/30)	5.17 (6/116)	1.31 (0.25, 6.84)/1	0.749

*N*: total number of subjects, *n*: positive number of subjects, OR: odds ratio, CI: confidence interval, article number: total number of the articles relevant to the association between vitiligo and HLA-A alleles, r: random effects model, and the others: fixed effects model.

**Table 5:** Association between vitiligo and human leukocyte antigen- (HLA-) A alleles in terms of typing methods.

Typing methods	Allele	Patients% (*n*/*N*)	Controls% (*n*/*N*)	OR (95% CI)/article number	*P*
Serological	A^*∗*^01	15.69 (91/580)	23.77 (444/1868)	0.95r (0.58, 1.56)/9	0.840
	A^*∗*^02	55.92 (373/667)	44.21 (1377/3115)	1.64r (1.26, 2.13)/11	<0.001
	A^*∗*^03	19.34 (118/610)	13.42 (322/2400)	1.58r (1.03, 2.42)/10	0.035
	A^*∗*^04	3.85 (2/52)	3.00 (3/100)	1.29 (0.21, 7.99)/1	0.782
	A^*∗*^09	20.07 (108/538)	26.25 (461/1756)	0.70 (0.54, 0.90)/8	0.005
	A^*∗*^10	10.56 (47/445)	12.79 (175/1368)	1.11r (0.51, 2.41)/7	0.786
	A^*∗*^11	17.99 (93/517)	14.61 (294/2012)	0.95 (0.71, 1.28)/9	0.751
	A^*∗*^19	22.50 (18/80)	43.75 (35/80)	0.19r (0.02, 2.02)/2	0.170
	A^*∗*^23	2.35 (2/85)	4.00 (8/200)	0.72 (0.17, 3.11)/2	0.662
	A^*∗*^24	25.00 (31/124)	48.12 (358/744)	0.79r (0.17, 3.76)/3	0.767
	A^*∗*^25	3.85 (2/52)	2.00 (2/100)	1.96 (0.27, 14.33)/1	0.507
	A^*∗*^26	10.48 (13/124)	18.28 (136/744)	0.71 (0.38, 1.32)/3	0.282
	A^*∗*^28	7.89 (31/393)	8.60 (109/1268)	0.74 (0.46, 1.17)/6	0.192
	A^*∗*^29	4.94 (12/243)	7.34 (89/1212)	0.78 (0.42, 1.44)/5	0.434
	A^*∗*^30	9.20 (24/261)	5.61 (63/1123)	1.55r (0.48, 5.03)/5	0.468
	A^*∗*^31	14.26 (15/105)	8.28 (90/1087)	2.26 (1.22, 4.19)/3	0.009
	A^*∗*^32	9.32 (11/118)	8.24 (53/643)	1.11 (0.55, 2.25)/3	0.774
	A^*∗*^33	3.03 (2/66)	2.58 (14/543)	1.23 (0.32, 4.77)/2	0.763
	A^*∗*^36	5.00 (2/40)	0.00 (0/40)	5.26 (0.24, 113.11)/1	0.289
	A^*∗*^38	0.00 (0/40)	2.50 (1/40)	0.33 (0.01, 8.22)/1	0.495
	A^*∗*^43	5.00 (2/40)	0.00 (0/40)	5.26 (0.24, 113.11)/1	0.289
	A^*∗*^66	0.00 (0/52)	2.00 (2/100)	0.38 (0.02, 7.96)/1	0.529
	A^*∗*^68	5.88 (5/85)	2.50 (5/200)	2.36 (0.67, 8.40)/2	0.317
	A^*∗*^80	5.00 (2/40)	10.00 (4/40)	0.47 (0.08, 2.75)/1	0.405
	Aw^*∗*^19	0.00 (0/48)	0.93 (1/107)	0.73 (0.03, 18.29)/1	0.849
	Aw^*∗*^23	2.08 (1/48)	4.67 (5/107)	0.43 (0.05, 3.82)/1	0.452
	Aw^*∗*^24	0.00 (0/48)	2.80 (3/107)	0.31 (0.02, 6.08)/1	0.439
	Aw^*∗*^30	20.83 (10/48)	28.04 (30/107)	0.68 (0.30, 1.52)/1	0.345
	Aw^*∗*^31	16.67 (8/48)	4.67 (5/107)	4.08 (1.26, 13.22)/1	0.019
	Aw^*∗*^32	4.17 (2/48)	8.41 (9/107)	0.47 (0.10, 2.28)/1	0.351
	Aw^*∗*^33	9.20 (8/87)	12.29 (80/651)	1.03 (0.46, 2.30)/2	0.944

Molecular	A^*∗*^01	52.15 (158/303)	47.07 (233/495)	0.94 (0.66, 1.33)/2	0.714
	A^*∗*^02	17.98 (388/2158)	17.74 (333/1877)	1.31r (0.84, 2.05)/4	0.238
	A^*∗*^03	21.55 (25/116)	23.05 (56/243)	0.92 (0.54, 1.56)/1	0.752
	A^*∗*^11	6.03 (7/116)	12.76 (31/243)	0.44 (0.19, 1.03)/1	0.058
	A^*∗*^23	7.76 (9/116)	9.88 (24/243)	0.77 (0.34, 1.71)/1	0.517
	A^*∗*^24	18.10 (21/116)	17.28 (42/243)	1.06 (0.59, 1.89)/1	0.849
	A^*∗*^25	13.20 (40/303)	6.46 (32/495)	0.71r (0.03, 19.23)/2	0.839
	A^*∗*^26	7.91 (163/2061)	11.41 (206/1805)	0.83r (0.50, 1.38)/3	0.479
	A^*∗*^29	8.62 (10/116)	10.70 (26/243)	0.79 (0.37, 1.69)/1	0.540
	A^*∗*^30	14.84 (50/337)	9.38 (56/597)	2.22r (0.72, 6.83)/3	0.166
	A^*∗*^31	3.35 (69/2061)	5.48 (99/1805)	0.68r (0.30, 1.55)/3	0.360
	A^*∗*^32	3.45 (4/116)	8.23 (20/243)	0.40 (0.13, 1.19)/1	0.100
	A^*∗*^33	22.84 (428/1874)	10.95 (170/1553)	1.56r (0.58, 4.24)/2	0.379
	A^*∗*^34	0.00 (0/116)	3.29 (8/243)	0.12 (0.01, 2.08)/1	0.145
	A^*∗*^36	0.00 (0/303)	2.42 (12/495)	0.12 (0.02, 0.91)/2	0.040
	A^*∗*^43	37.43 (70/187)	42.46 (107/252)	0.81 (0.55, 1.19)/1	0.289
	A^*∗*^66	3.30 (10/303)	8.89 (44/495)	0.70r (0.05, 10.81)/2	0.798
	A^*∗*^68	14.83 (278/1874)	13.33 (207/1553)	1.27r (0.76, 2.13)/2	0.363
	A^*∗*^69	0.00 (0/116)	0.82 (2/243)	0.41 (0.02, 8.71)/1	0.571
	A^*∗*^80	0.00 (0/116)	0.41 (1/243)	0.69 (0.03, 17.16)/1	0.823
	Aw^*∗*^19	0.74 (13/1758)	6.79 (89/1310)	0.10 (0.06, 0.18)/1	<0.001

*N*: total number of subjects, *n*: positive number of subjects, OR: odds ratio, CI: confidence interval, article number: total number of the articles relevant to the association between vitiligo and HLA-A alleles, r: random effects model, and the others: fixed effects model.
